# Knee bone tumors: findings on conventional radiology*

**DOI:** 10.1590/0100-3984.2013.0007

**Published:** 2016

**Authors:** Francisco Andrade Neto, Manuel Joaquim Diógenes Teixeira, Leonardo Heráclio do Carmo Araújo, Carlos Eduardo Barbosa Ponte

**Affiliations:** 1 Full Member of the Associação Brasileira de Oncologia Ortopédica (ABOO) and the Sociedade Brasileira de Ortopedia e Traumatologia (SBOT), Physician in the Unit of Orthopedics, Hospital Geral de Fortaleza (HGF), Fortaleza, CE, Brazil.; 2 Full Member of the Associação Brasileira de Oncologia Ortopédica (ABOO) and the Sociedade Brasileira de Ortopedia e Traumatologia (SBOT), Head of the Unit of Orthopedics, Hospital Geral de Fortaleza (HGF), Fortaleza, CE, Brazil.; 3 Full Member of the Sociedade Brasileira de Cirurgia do Joelho (SBCJ) and the Sociedade Brasileira de Ortopedia e Traumatologia (SBOT), Physician in the Unit of Orthopedics, Hospital Geral de Fortaleza (HGF), Fortaleza, CE, Brazil.; 4 Full Member of the Colégio Brasileiro de Radiologia e Diagnóstico por Imagem (CBR), Radiologist at the Hospital Monte Klinikum, Fortaleza, CE, Brazil.

**Keywords:** Neoplasms, bone tissue, Radiology/methods, Bone and bones/radiography, Orthopedics

## Abstract

The knee is a common site for bone tumors, whether clinically painful or not. Conventional radiology has been established as the first line of investigation in patients with knee pain and can reveal lesions that often generate questions not only for the generalist physician but also for the radiologist or general orthopedist. History, image examination, and histopathological analysis compose the essential tripod of the diagnosis of bone tumors, and conventional radiology is an essential diagnostic tool in patients with knee pain. This pictorial essay proposes to depict the main conventional radiography findings of the most common bone tumors around the knee, including benign and malignant tumors, as well as pseudo-tumors.

## Abstract (pt)

O joelho é sede comum de apresentação de tumores ósseos, sejam eles clinicamente dolorosos ou não. A radiologia convencional consagra-se como exame de primeira linha para investigação inicial dos doentes com dor no joelho e pode revelar lesões que muitas vezes geram dúvidas tanto para o médico generalista quanto para o radiologista ou ortopedista geral. História, exame de imagem e estudo histopatológico compõem o tripé essencial do diagnóstico dos tumores ósseos, sendo a radiografia simples o instrumento essencial de investigação diagnóstica nesses casos. Este ensaio iconográfico propõe expor os principais achados dos mais frequentes tumores ósseos, benignos, malignos e pseudotumorais do joelho.

## INTRODUCTION

The radiological diagnosis of bone tumors, often identified as incidental findings in asymptomatic patients, requires caution and evaluation by a specialist. For knee pain, conventional radiography is a complementary method of diagnosis that is essential to the investigation.

The fundamental elements for the differential diagnosis and evaluation of bone tumors using conventional radiography are patient history and age, together with the clinical presentation, anatomical location of the lesion, definition of the zone of transition between the lesion and host bone, and the radiographic characteristics of the lesion^(1,2)^. From these data, it is possible to establish differential diagnoses that are more precise, which can guide physicians in pursuing the investigation, carrying out staging and biopsy as necessary.

This study describes the main radiography findings of the most common bone tumors around the knee. All imaging diagnoses in this paper were confirmed by histopathological analysis.

## PSEUDOTUMORS

Pseudotumors are not categorized as true neoplasms, because they lack the specific pathological characteristics and contain no neoplastic cells. Pseudotumors frequently are the result of metabolic stimulation or hyperactivity of normal cells, such as osteoclasts.

### Simple bone cyst and aneurysmal bone cyst

**Figure 1:**
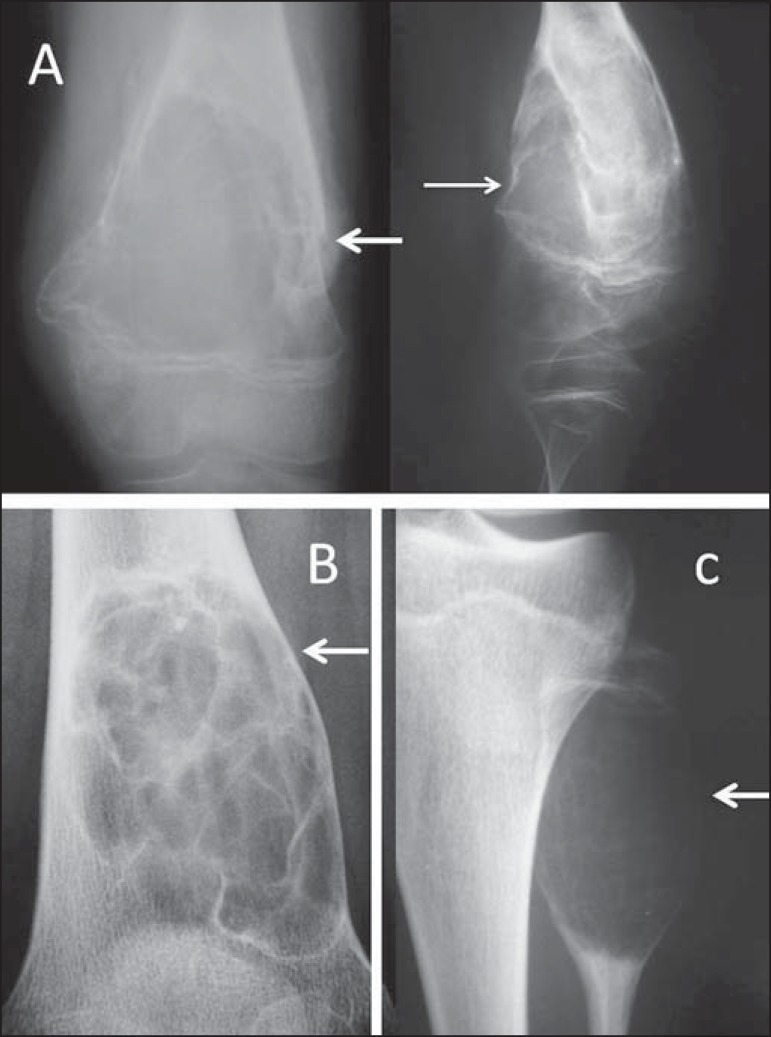
**A:** Conventional anteroposterior and lateral radiography of the left knee of a male schoolchild with knee pain. Extensive centric and metaphyseal lytic lesion expanding the cortical bone, with well-defined boundaries, a short transition zone, an adjacent sclerotic rim, and intralesional streaks of fibrotic bone, consistent with a simple bone cyst. There is a fracture with callus formation (arrows). **B:** Conventional radiography of the right knee of a male schoolchild shows the same findings, consistent with simple bone cyst. **C:** Extremely expansile, metaphyseal, lytic lesion, with a short transition zone and no sclerotic rim, with discrete intralesional streaks of fibrotic bone and not invading the growth plate, located in the proximal third of the left fibula (arrow) of an adolescent with knee pain. The findings are characteristic of an aneurysmal bone cyst.

A simple bone cyst of unknown cause is characterized by a well-defined, unicameral, radiolucent lesion, not breaking through the adjacent cortical bone, with a sclerotic rim^(1,2)^, as shown in Figures 1A and 1B.

Typically, simple bone cysts are metaphyseal lesions and appear during childhood or adolescence. Fracture occurs as the first clinical manifestation in up to 70% of cases^(1)^. The clinical diagnosis can be presumed with conventional radiography, but computed tomography (CT) and magnetic resonance imaging (MRI) allow for better lesion staging^(1,2)^.

With more striking radiographic features, aneurysmal bone cysts are painful metaphyseal lytic lesions which expand the adjacent cortex. They are fast growing and therefore have only a thin sclerotic rim or none at all. It is an expansile lesion with cavities containing hematic material from the trabecular bone, resulting in discrete intralesional streaks of fibrotic bone, which can be observed on conventional radiography (Figure 1C).

The anatomopathological study typically shows blood lacunae bordered by septa, osteoclasts with inflammatory infiltrate, and multinucleated giant cells, with no signs of malignancy.

### Fibrous dysplasia

Fibrous dysplasia is a benign fibro-osseous pseudotumor in which normal bone is replaced with fibrous tissue permeated with immature heterogeneous trabecular bone. It can be monostotic or polyostotic and affects the immature skeleton^(1,2)^.

Frequently asymptomatic, fibrous dysplasia can be diagnosed in adulthood as an incidental finding of gradual bone deformity on a radiological examination, or in childhood due to pathological fracture. With age, the lesion tends to gradually deform the affected long bones.

**Figure 2:**
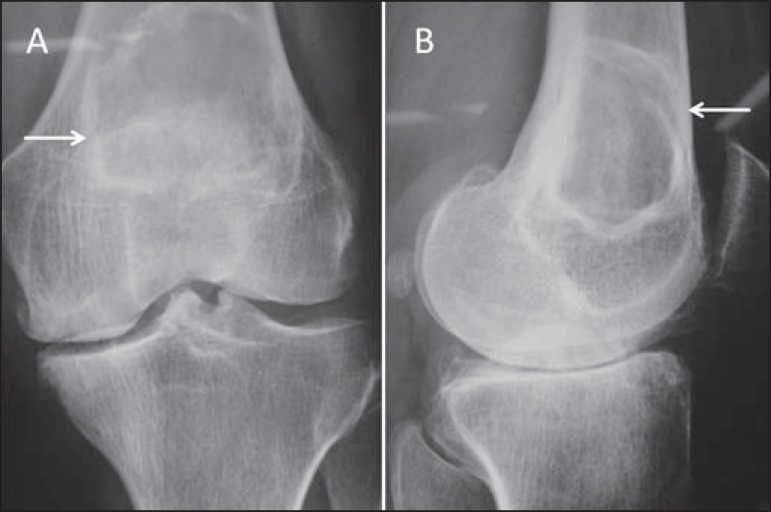
Conventional anteroposterior radiography **(A)** and conventional lateral radiography **(B)** of the left knee of an adult with mechanical knee pain. Well-defined, metaphyseal, lytic lesion on the distal femur, with an adjacent, reactive sclerotic rim (arrows), not breaking through the cortical bone. Characteristic "ground glass" aspect. There are also findings consistent with degenerative joint arthropathy, which may be the cause of the pain described by the patient.

Radiographic features include well-defined radiolucent lesions, with a characteristic intralesional ground-glass aspect and narrow zone of transition, frequently with a reactive sclerotic rim forming the so-called "ring sign" (Figure 2). The main differential diagnosis is simple bone cyst. Findings with conventional radiography are presumptive. Histopathological examination confirms the diagnosis.

### Bone infarction

Bone infarction has many causes, such as alcoholism, collagen diseases, glucocorticoid use, and blood diseases, and can affect all age groups^(1-3)^. The lesions are characterized by medullary bone necrosis and loss of normal bone trabeculae, leading to localized sclerosis.

Asymptomatic until it affects the joints, bone infarction is found upon investigation for adjacent articulation pain^(3)^. On conventional radiography, it can be difficult to differentiate bone infarction from enchondroma and chondrosarcoma. Bone scintigraphy is useful for determining whether the lesion metabolically active or not^(2,3)^. MRI is helpful in evaluating the extent of the lesion, as well as in conducting a detailed study of the adjacent articulation, and can reveal findings typical of osteonecrosis. Biopsies are reserved for cases in which the diagnosis is unclear or there is a need to exclude the possibility of a true neoplasm^(3)^.

**Figure 3:**
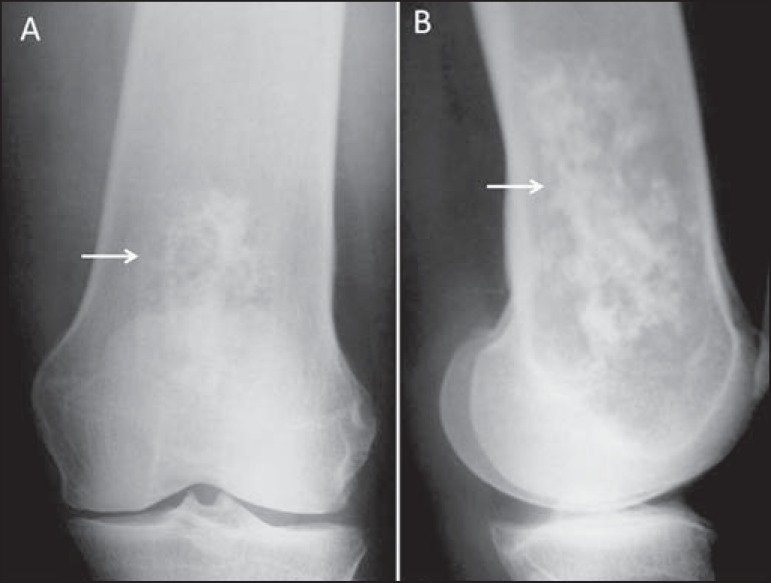
Conventional anteroposterior radiography **(A)** and conventional lateral radiography **(B)** of the left knee of a female patient with systemic lupus erythematosus and joint pain. The imaging shows a lesion with a mixed, heterogeneous radiological pattern in the medullary bone of the distal femur, with imprecise boundaries and a geographical aspect (arrows), although not breaking through the cortical bone. Tapered cortical bone can also be observed throughout the femur evaluated. Together with the clinical history, the MRI findings and the low concentration of radiopharmaceuticals on scintigraphy indicate bone infarction. The anatomopathological examination confirmed the diagnosis.

Conventional radiography shows focal or diffuse medullary osteosclerosis in one or more bones. Periarticular metaphyseal involvement is common. The lesions are mixed, with lytic areas permeated by areas of sclerosis, restricted to the bone marrow, and do not affect the cortex or induce periosteal reaction (Figure 3).

## BONE-FORMING TUMORS

### Osteosarcoma

Osteosarcoma is a malignant bone-forming tumor in which the mesenchymal neoplasm cells are capable of forming osteoid tissue or immature bone. The age distribution is bimodal, with the first peak of incidence in the second decade of life and the second after 50 years of age. Swelling and pain are the main complaints, which in general are followed by signs of inflammation and functional loss^(1-3)^.

**Figure 4:**
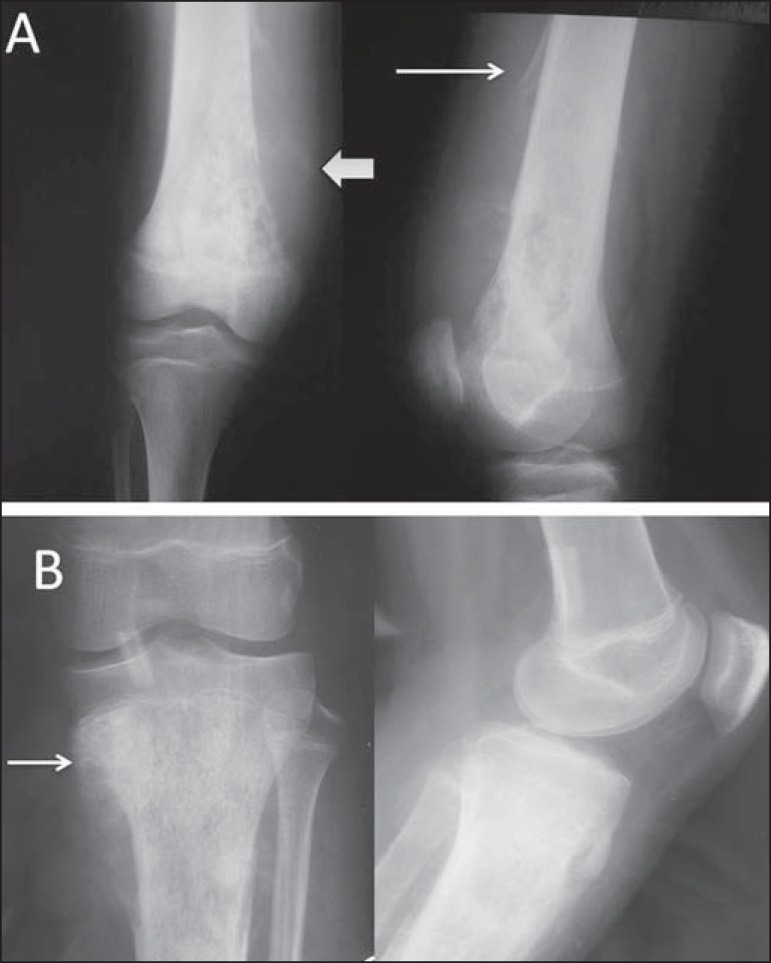
**A:** Anteroposterior and lateral conventional radiography of the right knee of a female adolescent, who presented with progressive pain and localized swelling, showing an aggressive mixed metaphyseal lesion, characterized by imprecise boundaries, Codman's triangle periosteal reaction (thin arrow), invasion of the cortex, and formation of a "bone outside the bone"aspect (thick arrow). **B:** Mixed metaphyseal lesion in the proximal left tibia, with a "sunburst" image (arrow) as well as a periosteal reaction resulting in the formation of Codman's triangle.

The radiologic manifestations of osteosarcoma can vary, depending on the histological subtype. The tumors can be purely lytic, such as the telangiectatic subtype, or totally sclerotic, such as the osteoblastic subtype. It is typically a metaphyseal lesion in an immature skeleton, with possible invasion of the growth plate^(1,2,4)^. In rare cases, there can be noncontiguous intramedullary lesions in the same bone, characteristic of skip metastasis, which denotes a worse prognosis^(1)^. In the more advanced stages, the tumor breaks through the cortical bone and invades adjacent tissues. At that stage, it induces a complex "sunburst" periosteal reaction showing up on conventional radiography as Codman's triangle and ossification in the soft tissues, giving the "bone outside the bone" appearance (Figure 4). Local and distant staging, followed by needle biopsy, are mandatory.

## CARTILAGE-FORMING TUMORS

### Osteochondroma

Frequently described as the most common benign tumor of the skeleton, osteochondroma can present as single or multiple lesions that are sessile or pedunculated, characteristic of hereditary multiple osteochondromatosis^(1-3,5)^. Patients normally do not feel pain but complain of a bony mass near a joint, most often the knee. In the specific case of the knee, osteochondroma can limit the range of movement and compress the peripheral nerves^(5)^.

**Figure 5:**
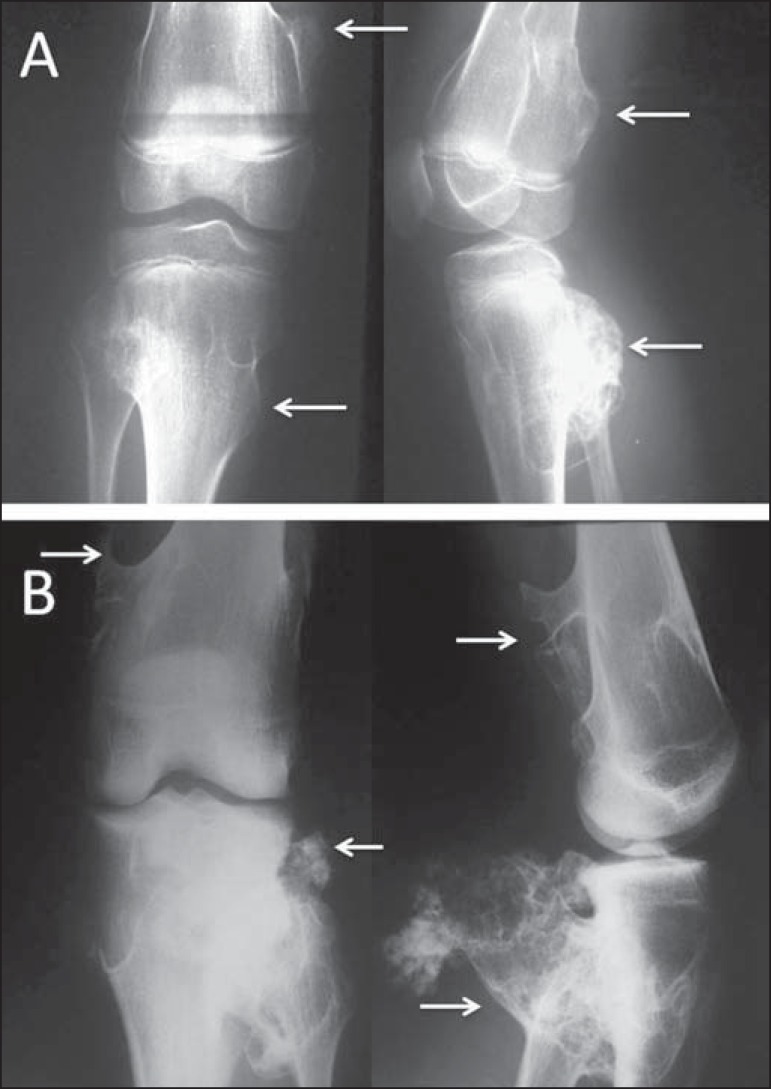
**A:** Patient with open physes, showing mixed sessile lesions in metaphyseal region of the distal femur and proximal tibia, with aspect of normal bone, characteristic of osteochondromas (arrows). **B:** Typical adult osteochondromas that tend to spread out from the joint. Note the extensive posterior lesion in the tibia and fibula (arrows), which causes pain by compression and limits joint mobility.

An osteochondroma is composed of normal bone and is covered by cartilage, typically metaphyseal (composed of cortical and medullary material) and centrifugal to the joint, without radiological signs of aggressiveness (Figure 5). An important radiological feature is the continuity between the cortex of the lesion and that of the host bone^(6)^. Osteochondromas grow from their cartilage cap, which is similar to a growth plate, and stop growing after skeletal maturation. If there is a volume increase after skeletal maturity, further diagnostic investigation should be conducted to exclude the possibility of sarcomatous transformation. Transformation to chondrosarcoma is rare, occurring in ≤ 5% of all cases of multiple osteochondromatosis^(5,7)^.

### Enchondroma

**Figure 6:**
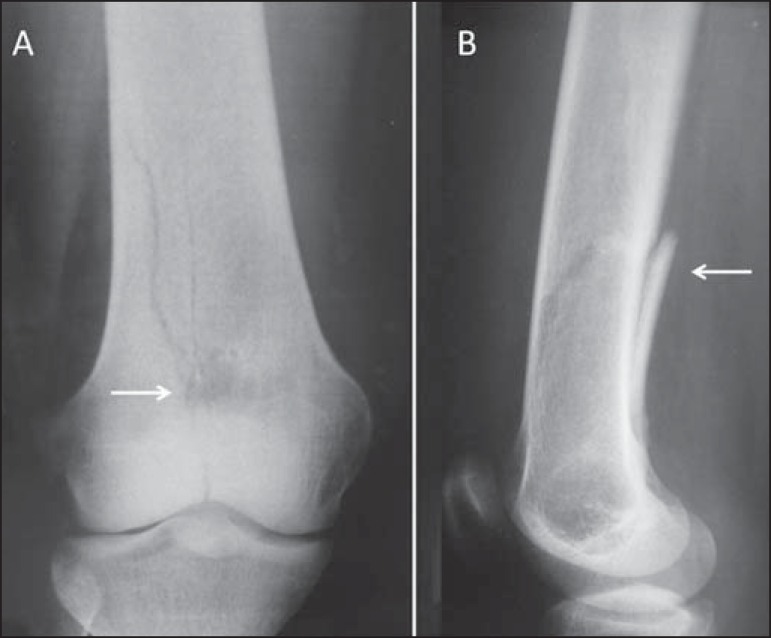
A previously asymptomatic young adult with a pain episode and acute limitation of right knee mobility after a common trauma. Anteroposterior conventional radiography of the knee (**A**) showing a small, rounded lytic lesion, with small intralesional foci of calcification, precise boundaries, and no sclerotic rim, in the distal femur (arrow), accompanied by evidence of a metaphyseal fracture (arrow in **B**) centered on the lesion, extending to the joint, characterizing a pathological bone fracture.

An enchondroma is a benign tumor characterized by the formation of mature hyaline cartilage tissue^(1)^. It can present as single or multiple lesions. It is often asymptomatic and typically affects the bones of the hand, the bones of the foot, or the proximal femur. In the knee, enchondroma can be an incidental finding of an ancillary examination performed because of other complaints from the patient or when associated with complications such as pathological fracture^(1,2)^, as depicted in Figure 6.

An enchondroma is lytic and ovoid, with foci of intralesional calcification and no adjacent sclerotic rim. In the bones of the hand, it can be expansile, although it always remains within the borders of the cortical bone. Diagnostic workup with MRI and CT is useful in cases of diagnostic uncertainty and to confirm intralesional calcification^(8)^.

### Chondroblastoma

Also known as Codman's tumor, chondroblastoma is a benign yet aggressive cartilage-forming bone tumor that affects the epiphysis in immature skeletons. The clinical presentation includes joint pain and localized swelling in young patients with an open growth plate. The distal tibia, proximal femur, and proximal humerus are the most common locations^(1-3)^.

**Figure 7:**
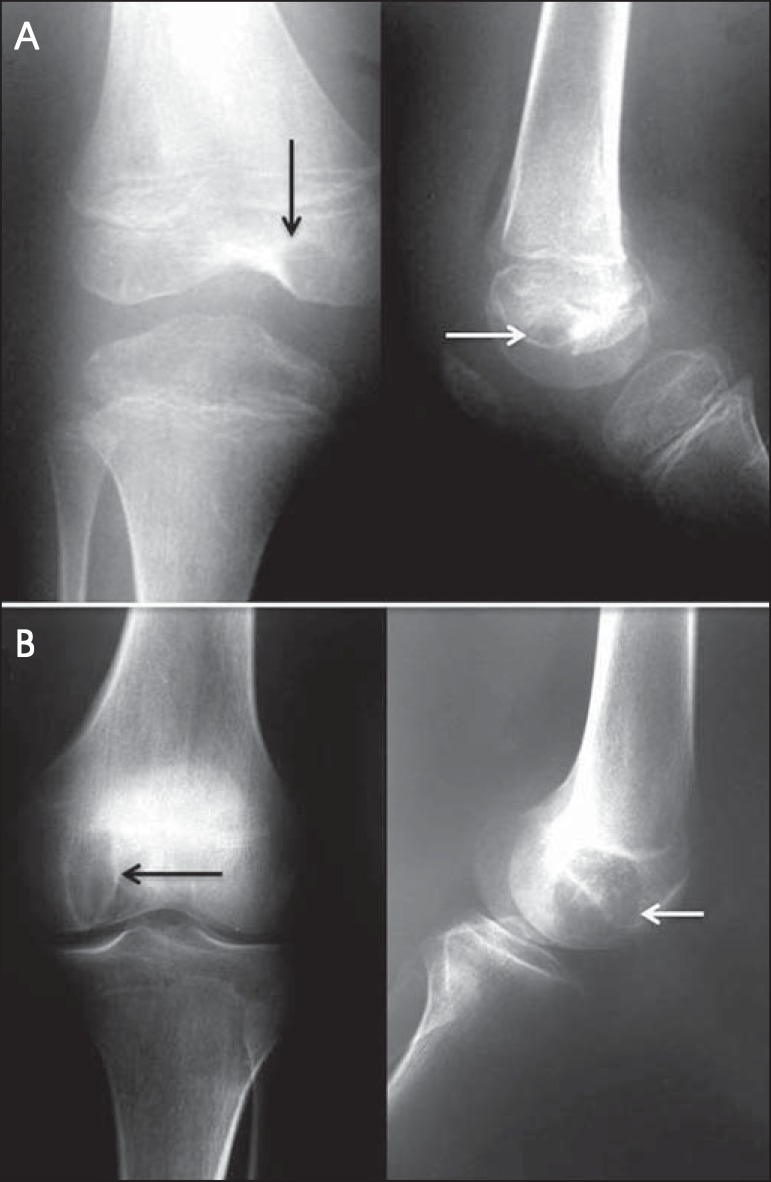
**A:** A 10-year-old patient with progressive pain and swelling of the right knee. Black and white arrows indicate an oval-shaped, osteolytic epiphyseal lesion with a sclerotic rim, not breaking through the cortex, with intralesional foci of calcification, in the medial condyle of the femur. There are also signs of joint effusion characterized by distension of the suprapatellar bursa. **B:** A 17-year-old adolescent, showing closed physes and the same clinical complaints. Black and white arrows mark the lesion with the same radiological characteristics.

Radiography of a chondroblastoma (Figure 7) shows well-defined osteolytic epiphyseal lesions, with a narrow zone of transition, a reactive sclerotic rim which generally does not break through the cortex, and foci of calcification within the lesion, characteristic of the cartilaginous origin of this benign tumor.

### Chondromyxoid fibroma

Chondromyxoid fibroma is an aggressive benign cartilaginous bone tumor, with proliferation of myxoid and fibrous tissues. It accounts for 0.5% of all primary bone tumors and can affect all age groups, although it is most common among adolescents and young adults^(1)^.

The clinical presentation of chondromyxoid fibroma consists of progressive pain in the affected segment, local swelling, and (in some cases) other signs of inflammation.

**Figure 8:**
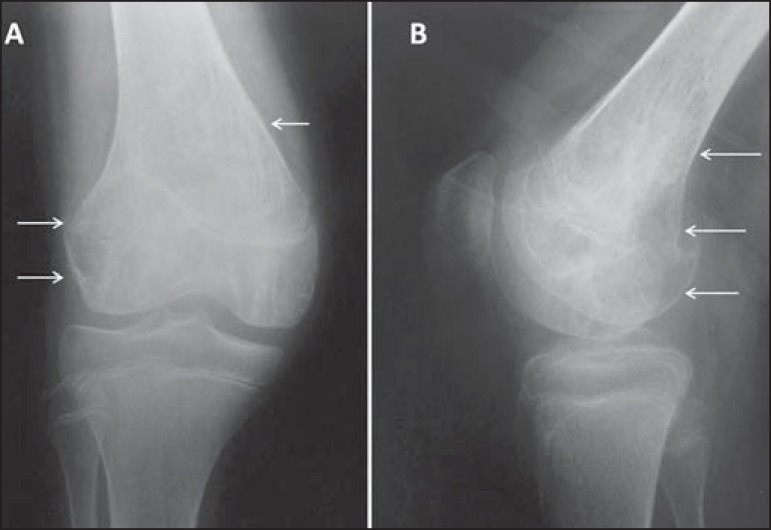
A 14-year-old patient with progressive pain and localized swelling in the right knee. Anteroposterior and lateral radiological studies (**A** and **B**, respectively), showing a metaphyseal lytic lesion in the distal femur, extending to the epiphysis, with a discrete, reactive sclerotic rim, and breaking through the cortex in the lateral condyle of the femur (arrows).

Although the radiographic features of chondromyxoid fibroma can vary, it often presents as a metaphyseal, lytic, eccentric, expansile lesion, with a narrow zone of transition and a reactive sclerotic rim. Foci of intralesional calcification are uncommon (Figure 8). The differential diagnosis mainly includes simple aneurysmal bone cyst and, in some cases, giant cell tumor. In case of uncertainty regarding the possibility of a more aggressive lesion, staging and biopsy constitute the safest course of action^(1-3)^.

### Chondrosarcoma

Chondrosarcoma is a malignant cartilaginous tumor, described as one of the most common primary malignant bone tumors, second only to multiple myeloma. It comprises a heterogeneous group of lesions with morphological factors and biological behavior that range from non-metastatic, slow-growing lesions to highly aggressive lesions with early metastatic dissemination^(1,3,4)^.

Chondrosarcoma predominantly affects males, often after the fifth decade of life, and is rare among young individuals. The most common site is the hip, followed by the femur, including the area around the knee, and the humerus^(1,3,4)^.

**Figure 9:**
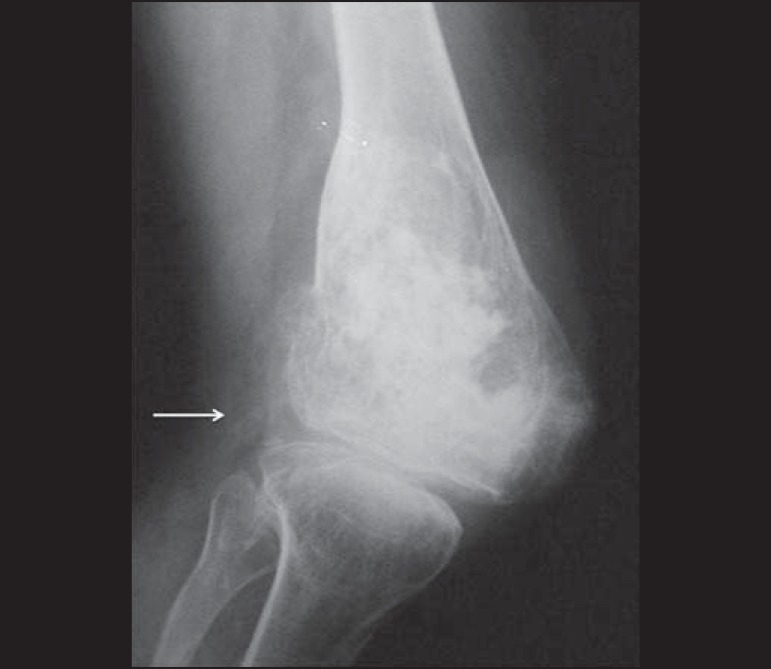
Radiograph of an adult with knee pain showing an aggressive, mixed metaphyseal lesion in the distal femur, with various foci of confluent calcification. The lesion is breaking through the cortex, with calcification also in the soft tissues (arrow).

The radiographic features of chondrosarcoma include osteolytic lesions with signs of local aggressiveness and soft tissue masses with calcification (Figure 9). The treatment is basically surgical, given that the majority of these tumors do not respond to chemotherapy or radiotherapy^(1-4)^.

## MEDULLARY TUMORS

### Ewing's sarcoma

Ewing's sarcoma is a small round-cell tumor, arising in the bone marrow, that occurs predominantly in the long bones of patients with an immature skeleton. It accounts for approximately 12% of all malignant tumors, most frequently affecting patients ≤ 15 years of age, without a predilection for males or females^(1)^. The area around the knee is affected in up to 10% of cases^(1,3)^. It is the third most frequent among bone sarcomas, after osteosarcoma and chondrosarcoma^(1-3)^.

Ewing's sarcoma is a very aggressive lesion, the main complaints being intense pain and swelling of the affected segment, localized signs of inflammation, and systemic symptoms such as weight loss, adynamia, and fever^(2)^.

**Figure 10:**
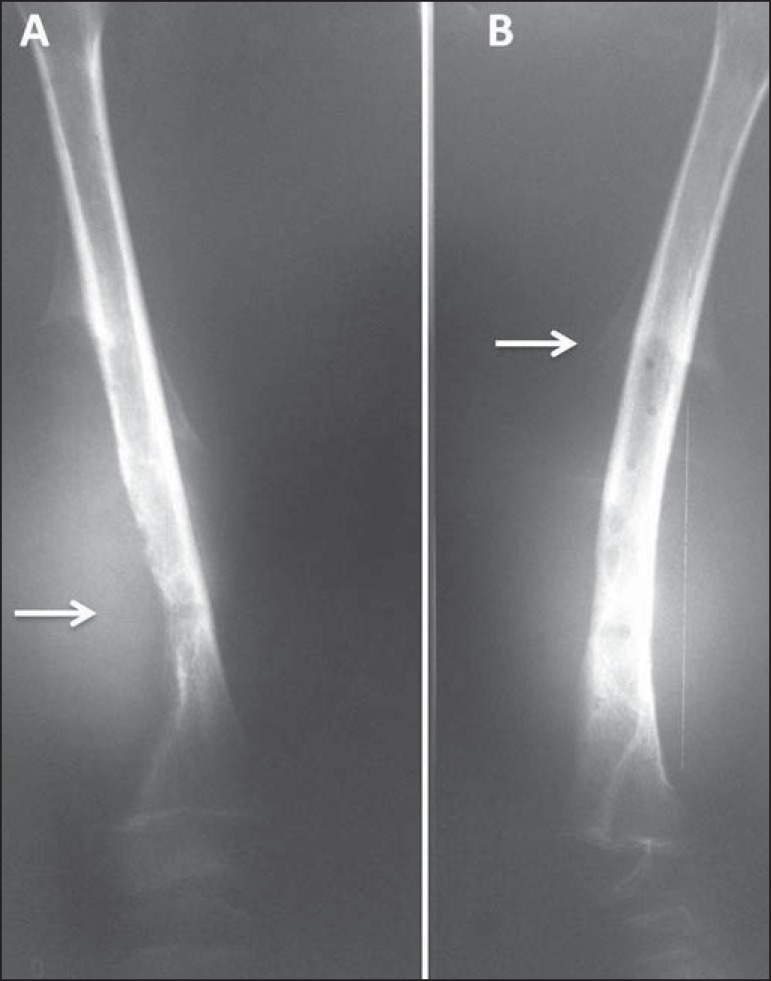
A 9-year-old male patient with pain and a large mass in the right thigh. **A:** Anteroposterior radiography of the left femur showing an essentially lytic metadiaphyseal lesion (arrow), an undefined zone of transition, increased density in the soft tissues, and destruction of the bone cortex. **B:** The same characteristics are seen with the formation of a Codman's triangle periosteal reaction (arrow). Radiologically aggressive lesion.

On radiographs, Ewing's sarcoma typically presents as a radiolucent, ill-defined infiltrative lesion located on the diaphysis of long bones, inducing a typical "onion-skin" periosteal reaction, and frequently produces large soft tissue masses without foci of calcification^(1-3,9,10)^, as shown in Figure 10.

The differential diagnosis of Ewing's sarcoma includes osteomyelitis, eosinophilic granuloma, lymphoma, neuroblastoma metastasis, leukemia, and, in some cases, telangiectatic osteosarcoma, which appears as an essentially lytic lesion on conventional radiography.

In patients with radiologically aggressive Ewing's sarcoma, staging (local and systemic) is mandatory and should precede biopsy. An MRI scan is quite helpful in local staging and in evaluating the involvement of the soft tissues^(9)^. As for osteosarcomas, the treatment protocol consists of neoadjuvant chemotherapy, followed by surgery and adjuvant chemotherapy^(1-3)^.

### Multiple myeloma

Multiple myeloma is the most common primary bone neoplasm. Most cases occur in patients in the fifth or sixth decade of life. Clinically, the initial complaint is bone pain, often generalized, together with pallor and alterations in kidney function in the more advanced stages. From a biochemical point of view, severe anemia can be seen, as can an increase in erythrocyte sedimentation rate, serum protein electrophoresis showing a monoclonal spike in the gamma-globulin fraction. Plasmacytic hyperplasia on myelography confirms the diagnosis^(1-4)^.

**Figure 11:**
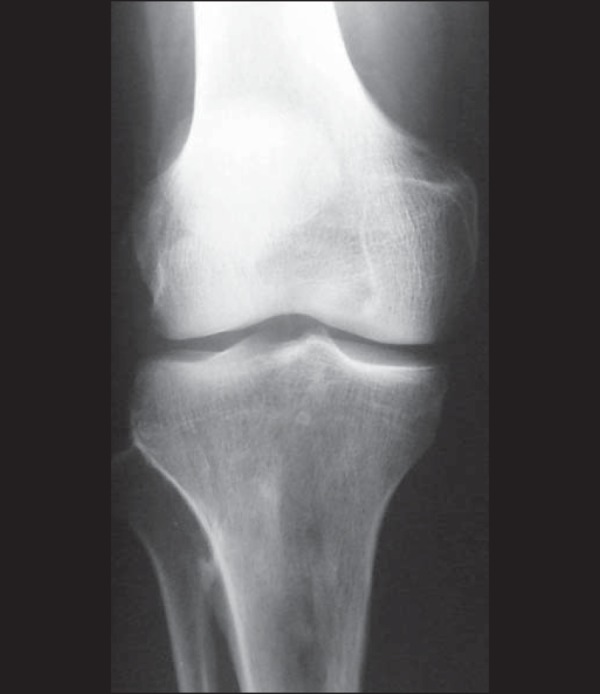
A 50-year-old patient with knee pain and radiological findings indicating an aggressive lesion. Anteroposterior radiography of the knee, showing tapering of the cortical bone in the proximal tibia, presence of a mottled, mixed medullary lesion with an imprecise zone of transition, extending from the subchondral boné to the diaphysis. The main differential diagnosis is metastasis, hyperparathyroidism, and lymphoma. Laboratory tests confirmed the diagnosis of multiple myeloma.

As exemplified in Figure 11, the radiologic findings in multiple myeloma include diffuse osteopenia accompanied by osteolytic lesions with the typical punched-out aspect, an endosteal lesion, a broad zone of transition (sometimes ill-defined, with imprecise borders), and no periosteal reaction; the condition can evolve to pathological fracture, with accentuation of localized acute pain^(1,2)^.

Plasmacytoma is a tumor that is histologically identical to multiple myeloma, although it is localized, without systemic repercussions or alterations on protein electrophoresis. It can be diagnosed only through biopsy^(1-3)^.

## OTHER CONNECTIVE TISSUE TUMORS

### Non-ossifying fibroma

**Figure 12:**
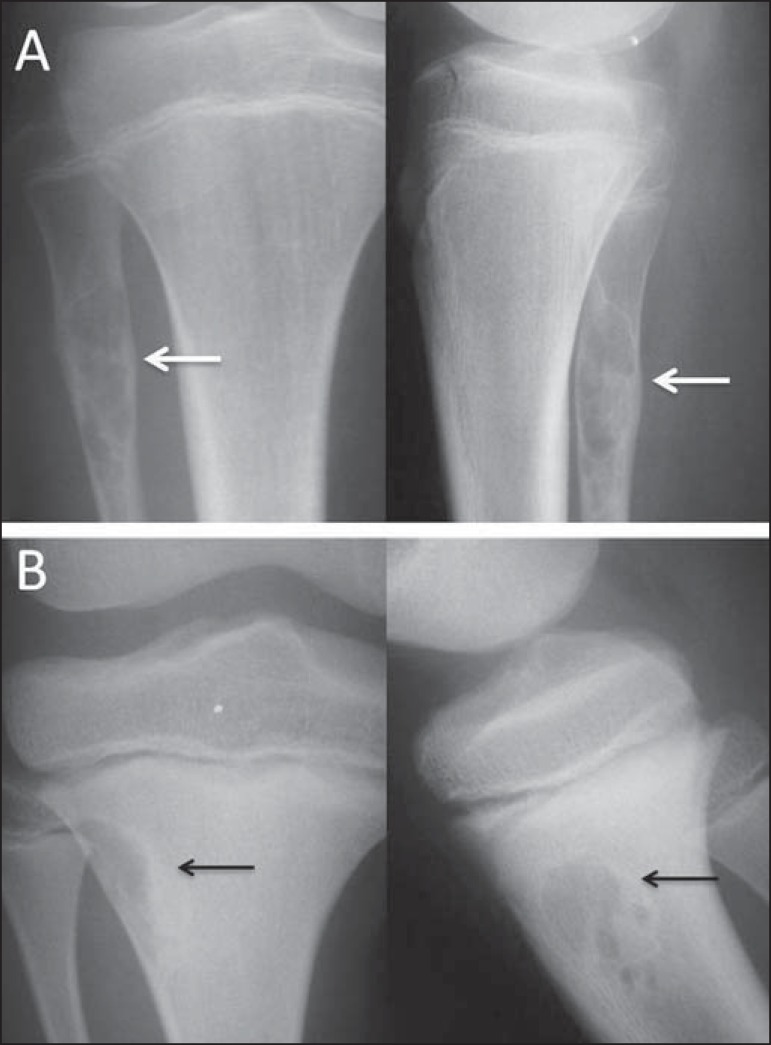
**A:** Anteroposterior and lateral radiography of an adolescent who suffered direct trauma on the knee region. Well-circumscribed lytic lesion (arrows) in the metaphyseal region of the fibula, not breaking through the cortex but extending to the bone marrow, with a sclerotic rim. Painless lesion, consistent with nonossifying fibroma. **B:** Lesion with similar characteristics (arrows) observed in the anterolateral region of the proximal tibia; the lesion is smaller and more wellcircumscribed in the cortical bone, not extending to the bone marrow, and is therefore designated a fibrous cortical defect. The lesions are histologically identical and do not show radiological characteristics indicative of aggressive lesions.

Non-ossifying fibroma affects children and teenagers and is characterized by juxtacortical radiolucent lesions, well-circumscribed by a sclerotic rim, without breaking through the cortical bone, without a periosteal reaction, and extending to the bone marrow. Although the fibrous cortical defect is histologically identical to that of ossifying fibroma, the radiological features vary, because the lesion does not reach the bone marrow, being restricted to the cortex, near the growth plate (Figure 12). Non-ossifying fibroma occurs in ≤ 30% of the population. Some authors use the term fibroxanthoma for both^(1,6)^. The lesions are asymptomatic and tend to calcify with age. They can be multiple or isolated, as well as monostotic or polyostotic^(1-3)^. The differential diagnosis includes fibrous dysplasia, simple bone cyst, and even chondromyxoid fibroma. In some cases, non-ossifying fibroma is discovered as an incidental finding, often on MRI scans obtained for the investigation of meniscal or ligament tears.

The radiological findings of non-ossifying fibromas are quite characteristic, and bone biopsy for diagnostic confirmation is rarely necessary^(1-3)^.

## METASTASES

**Figure 13:**
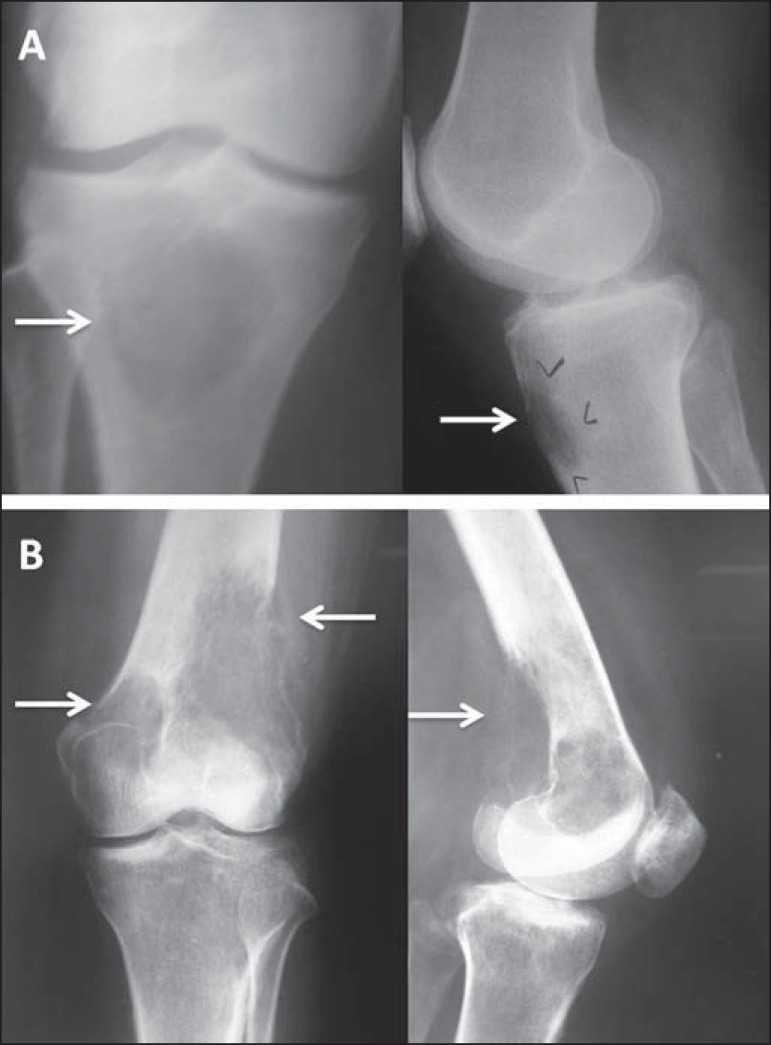
**A:** Anteroposterior and lateral radiography of the right knee of a female patient with a primary breast tumor. Metaphyseal lytic lesion on the tibia, with an imprecise zone of transition and ill-defined borders (arrows). **B:** Another case of a female patient with a primary breast tumor, who presented with knee pain. The image shows radiological features of aggressiveness, characterized by lytic lesion on the distal left femur, which breaks through the cortical bone (arrows), invades the adjacent soft parts, without a sclerotic rim and with an imprecise zone of transition. There are also lesions in the medial condyle of the fêmur and proximal third of the tibia.

Frequently, bone tissue is the site of a metastatic lesion, which, in addition to indicating a worse prognosis, can evolve to pathological fracture and can worsen the quality of life and treatment of the patient^(1-4)^. Bone metastases can present with osteolytic, osteoblastic, or mixed patterns^(2-4)^. The presentation varies and frequently surprises radiologists and surgeons (Figure 13).

When a metastasis is aggressive, it is essential that careful local and systemic staging be carried out, including ancillary tests such as CT, MRI, and scintigraphy^(1,3,9)^. If the primary site is not identified during staging, a lesion biopsy is fundamental for the histopathological and immunohistochemical evaluations^(6)^.

## GIANT CELL TUMOR OF BONE

Giant cell tumors of bone comprise a special group of bone tumors, which do not form bone or cartilage but simply promote osteoclast-mediated bone resorption, and can also be referred to as osteoclastomas. A giant cell tumor of bone is considered an aggressive benign tumor that rarely metastasizes. It usually affects patients in the third or fourth decades of life, and the most common sites are the distal femur, proximal tibia, and proximal humerus^(1,2,6,11)^.

**Figure 14:**
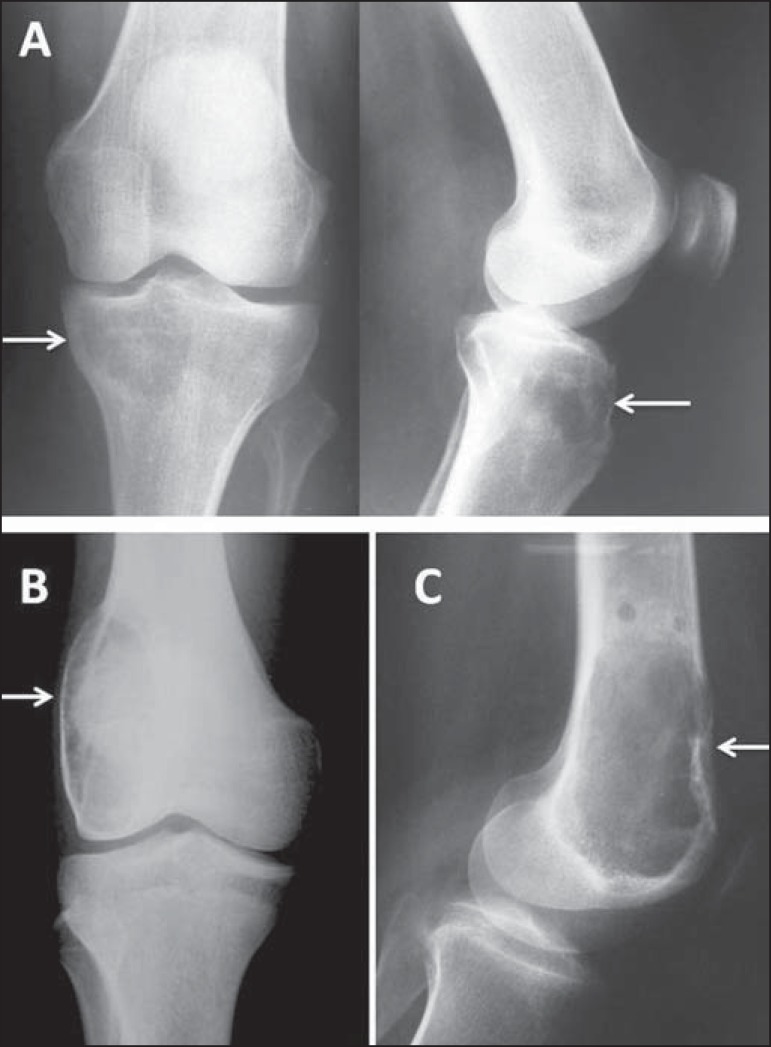
**A:** Adult female patient with recent knee pain; radiography shows an eccentric epiphyseal lytic lesion in the medial condyle of the tibia, without a sclerotic rim, with a narrow zone of transition, and without a periosteal reaction or invasion of soft tissues (arrows). **B:** Radiography of another patient with closed physes, showing a painful epiphyseal eccentric lytic lesion that expands and thins the cortical bone in the lateral condyle of the femur (arrow), extending to the subchondral bone. **C:** Lateral radiography of the knee in another case, with an epiphyseal lytic lesion that thins and breaks through the anterior cortical bone. All findings are consistent with a giant cell tumor in distinct phases of evolution.

Clinically, the complaints of patients with giant cell tumor of bone include pain, localized swelling, and functional impairment. The radiological aspect is fairly characteristic, including an eccentric epiphyseal lytic lesion with metaphyseal extension, in skeletally mature patients, without a sclerotic rim, frequently with cortex rupture and invasion of the articular or soft parts^(1,2,11)^, as depicted in Figure 14.

The differential diagnosis of giant cell tumor of bone includes mainly aneurysmal bone cyst and the brown pseudo-tumor seen in hyperparathyroidism. Local and systemic staging is recommended. Needle biopsy completes the diagnostic process^(11,12)^.

## CONCLUSION

The study of bone tumors is a challenge. The attending physician and radiologist should both be aware of the clinical characteristics of the patients being investigated and the radiographic features of the lesions. The characteristics of the lesions seen on conventional radiography can define the differential diagnosis, resulting in an appropriate clinical assessment.
